# A high-density genetic map of extra-long staple cotton (*Gossypium barbadense)* constructed using genotyping-by-sequencing based single nucleotide polymorphic markers and identification of fiber traits-related QTL in a recombinant inbred line population

**DOI:** 10.1186/s12864-018-4890-8

**Published:** 2018-06-25

**Authors:** Liping Fan, Liping Wang, Xinyi Wang, Haiyan Zhang, Yanfei Zhu, Jiayan Guo, Wenwei Gao, Hongwei Geng, Quanjia Chen, Yanying Qu

**Affiliations:** Department of Agronomy, Key Laboratory of Agriculture Biological Technology, Xinjiang Agriculture University, Urumqi, 830052 China

**Keywords:** *Gossypium barbadense*, Genotyping-by-sequencing (GBS), Fiber quality traits, Lint yield, Quantitative trait locus

## Abstract

**Background:**

*Gossypium barbadense* (Sea Island, Egyptian or Pima cotton) cotton has high fiber quality, however, few studies have investigated the genetic basis of its traits using molecular markers. Genome complexity reduction approaches such as genotyping-by-sequencing have been utilized to develop abundant markers for the construction of high-density genetic maps to locate quantitative trait loci (QTLs).

**Results:**

The Chinese *G. barbadense* cultivar 5917 and American Pima S-7 were used to develop a recombinant inbred line (RIL) population with 143 lines. The 143 RILs together with their parents were tested in three replicated field tests for lint yield traits (boll weight and lint percentage) and fiber quality traits (fiber length, fiber elongation, fiber strength, fiber uniformity and micronaire) and then genotyped using GBS to develop single-nucleotide polymorphism (SNP) markers. A high-density genetic map with 26 linkage groups (LGs) was constructed using 3557 GBS SNPs spanning a total genetic distance of 3076.23 cM at an average density of 1.09 cM between adjacent markers. A total of 42 QTLs were identified, including 24 QTLs on 12 LGs for fiber quality and 18 QTLs on 7 LGs for lint yield traits, with LG1 (9 QTLs), LG10 (7 QTLs) and LG14 (6 QTLs) carrying more QTLs. Common QTLs for the same traits and overlapping QTLs for different traits were detected. Each individual QTLs explained 0.97 to 20.7% of the phenotypic variation.

**Conclusions:**

This study represents one of the first genetic mapping studies on the fiber quality and lint yield traits in a RIL population of *G. barbadense* using GBS-SNPs. The results provide important information for the subsequent fine mapping of QTLs and the prediction of candidate genes towards map-based cloning and marker-assisted selection in cotton.

## Background

Genetic maps play an important role in evaluating the evolution of cotton chromosomes and the discovery of *Gossypium* genetic resources to improve the world’s most prominent natural fiber [1]. Previous quantitative trait locus (QTLs) studies on fiber and lint yield traits involved diploid cotton populations [2–6], upland cotton populations [7–12] and interspecific populations [13–16], although few studies have been conducted on intraspecific populations of *Gossypium barbadense* to identify fiber and lint yield QTLs [17, 18]. Previous studies of fiber quality and lint yield traits were primarily based on molecular markers with low polymorphic rates [19–21]. The genetic maps were severely unsaturated and contained large gaps, leading to fine mapping of the QTLs underlying the traits of interest difficult. Without increased numbers and improved types of markers, a high-density genetic map cannot be constructed. Recently, with the development of genetic markers, single-nucleotide polymorphism (SNP) markers rich in polymorphisms were found to be distributed throughout the cotton genome. Many complexity reduction approaches are available, such as genotyping-by-sequencing (GBS) [22–24], restriction site-associated DNA (RAD) sequencing [25–29] and specific locus-amplified fragment (SLAF) sequencing [30–34]. These technologies have been applied to many crops to construct genetic maps for QTL mapping.

Diouf et al. [22] constructed an ultra-high-density genetic map of intraspecific upland cotton using GBS technology that spanned 4768.098 cM, with an average distance of 0.92 cM, and contained 5178 SNP markers. Wang et al. [29] used RAD technology to construct a genetic map of intraspecific *Gossypium hirsutum*. The map included 4153 loci and spanned 3500 cM, with an average distance between two adjacent markers of 0.83 cM. Zhang et al. [33] constructed an intraspecific high-density genetic map of upland cotton using SLAF sequencing technology. The resulting genetic map contained 5521 markers spanning 3259.37 cM, and the average distance between adjacent markers was 0.78 cM. However, a high-density map within *G. barbadense* is currently unavailable.

In this study, a recombinant inbred line (RIL) population consisting of 143 accessions was constructed using Chinese *G. barbadense* 5917 and American Pima S-7 as parents. The genetic map using GBS was constructed in reference to the scaffold map of *G. barbadense* [35]. A total of 3557 SNP markers were used to construct a high-density genetic map for detection of QTLs based on fiber quality and yield trait data collected over 3 years.

## Results

### Analysis of fiber trait performance in the RIL population

The two parents exhibit unique characteristics with regard to fiber quality traits. The population derived from the parental strains had transgressive inheritance traits and characteristics with normal distributions. A distinct separation was observed among the populations despite the small differences between the two parents.

The results of one-way ANOVA showed no significant differences in seven traits between the two parents except for fiber strength (FS) and lint percentage (LP) (Table 1). The maximum and minimum values in the population were higher than those in the parents, indicating transgressive inheritance. The absolute values of kurtosis and skewness were all < 1, except for the kurtosis values for micronaire (MIC) and LP, which were 1.04 and 1.02, respectively. The fiber elongation (FE) and LP showed little difference between the parents, although large variations in these traits were observed in the progeny because of interactions between genes.

**Table 1:** The results of the overall analysis of parental and population data

Traits	Parents			Population				
	5917	PimaS-7	Range	MIN	MAX	MEAN	Kurt	Skew
FL	35.68	34	1.68	32.53	40.13	35.51	0.52	0.3
FU	88.52	87.31	1.21	84.51	90.3	87.64	−0.19	−0.25
FS	46.84	39.21	7.64*	33.38	54.85	43.9	0.81	−0.13
FE	6.18	6.26	−0.08	5.12	8.1	6.36	−0.25	0.27
MIC	4.11	4.39	−0.28	3.22	4.9	4.2	1.04	−0.08
BW	3.12	2.95	0.17	2.14	4.68	3.49	−0.04	−0.11
LP	0.33	0.38	−0.05*	0.29	0.47	0.36	1.02	2.86

*significance at *P* = 0.05

Correlations were detected among the seven traits (Table 2). Positive correlations were detected for fiber length (FL) with FS and fiber uniformity (FU); FS with MIC; FU with boll weight (BW). Negative correlations were detected between FL and FE, FE and BW, and FU and LP.

**Table 2:** The correlation analysis between seven fiber traits

	FL	FS	MIC	FU	FE	BW	LP
FL	1						
FS	.273**	1					
MIC	−.163	.242**	1				
FU	.541**	.440**	−.037	1			
FE	−.429**	−.149	.131	−.055	1		
BW	.94	.243**	.153	.174*	−.173*	1	
LP	−.104	−.027	.165	−.177*	−.19	−.127	1

*significance at *P* = 0.05

**significance at *P* = 0.1

The broad-sense heritability values ranged from 0.66–0.98. FL, FS and MIC had values of 0.73, 0.74 and 0.68, respectively (Table 3). LP had the highest broad-sense heritability values among all traits, indicating that it was primarily controlled by genotype.

**Table 3:** ANOVA for seven fiber traits and a broad-sense heritability in RIL population

Factor	DF	Sum of squares						
		FL	FU	MIC	FE	FS	BW	LP
Gen	142	4.29**	2.82**	0.22**	0.69**	21.72**	0.53**	0.021**
Env	2	19.9**	115.89**	9.44**	31.90**	365.61**	9.23**	0.00322**
Gen*Env	256	2.31**	1.99**	0.14**	0.40**	12.14**	0.29**	0.0008**
Broad-sense heritability		.073	0.66	0.68	0.68	0.74	0.74	0.98

*, ** Significant differences at *P* = 0.05 or *P* = 0.1, respectively

### Analysis of GBS data and markers

The clean base data for cultivar 5917 were 2.262 G, and the GC content was 38.3%. The clean base data for cultivar PimaS-7 were 2.656 G, and the GC content was 37.78%. In this study, the scaffold of *G. barbadense* was used as the reference genome. Cultivars 5917 and PimaS-7 yielded 7,785,604 and 9,127,821 reads, respectively, and the average sequencing depth was 24.54. A total of 175,365 polymorphic SNP markers were detected between 5917 and PimaS-7.

After filtering out missing sites in the parental information, 175,365 SNP markers were found, and 60,003 (34.22%) of which were polymorphic among whole markers. Of these, 15,891 SNP markers could be used for further analysis of the RIL population. No abnormal bases were found, indicating that the genotyping accuracy was high. These markers were filtered at an integrity standard of 65%, and 12,848 markers remained. After filtering using a *P*-value threshold of 0.001 and the k-nearest neighbor algorithm, 3557 markers were obtained to construct the genetic map, and the QTLs were analyzed.

### Quality assessment of high-throughput markers

Of the 3557 markers, 787 were segregation distortion markers (*P* < 0.05), and they were distributed among 24 linkage groups. Linkage groups LG8 and LG26 had no significant segregation distortion markers. The number of markers in the 24 linkage groups was unevenly distributed, with 352 markers in the A subgenome and 435 markers in the D subgenome. LG15 had the most markers (125), while LG12 and LG18 contained only two (Table 4). Eighty-one segregation distortion regions (SDRs) were distributed among 18 linkage groups. The A subgenome harbored 35 SDRs, and the D subgenome contained the rest.

**Table 4:** The significant of segregation distortion marks among 26 linkage groups

A subgenome	Marker	SDM	SDR	Rate	D subgenome	Marker	SDM	SDR	Rate
LG1	193	29	3	15%	LG14	279	21	2	8%
LG2	550	30	1	5%	LG15	129	125	13	97%
LG3	56	24	3	43%	LG16	39	13	0	33%
LG4	126	9	1	7%	LG17	45	11	0	24%
LG5	58	57	8	98%	LG18	50	2	0	4%
LG6	50	22	2	44%	LG19	38	25	1	66%
LG7	287	86	8	30%	LG20	88	12	0	14%
LG8	119	none	0	0%	LG21	131	62	8	47%
LG9	42	12	2	29%	LG22	85	76	8	89%
LG10	372	37	4	10%	LG23	29	5	0	17%
LG11	117	38	2	32%	LG24	42	39	7	93%
LG12	479	2	0	0%	LG25	68	44	7	65%
LG13	51	6	1	12%	LG26	34	none	0	0%
total		352	35				435	46	

### Construction of genetic maps based on the *G. barbadense* reference genome

**Fig. 1:**
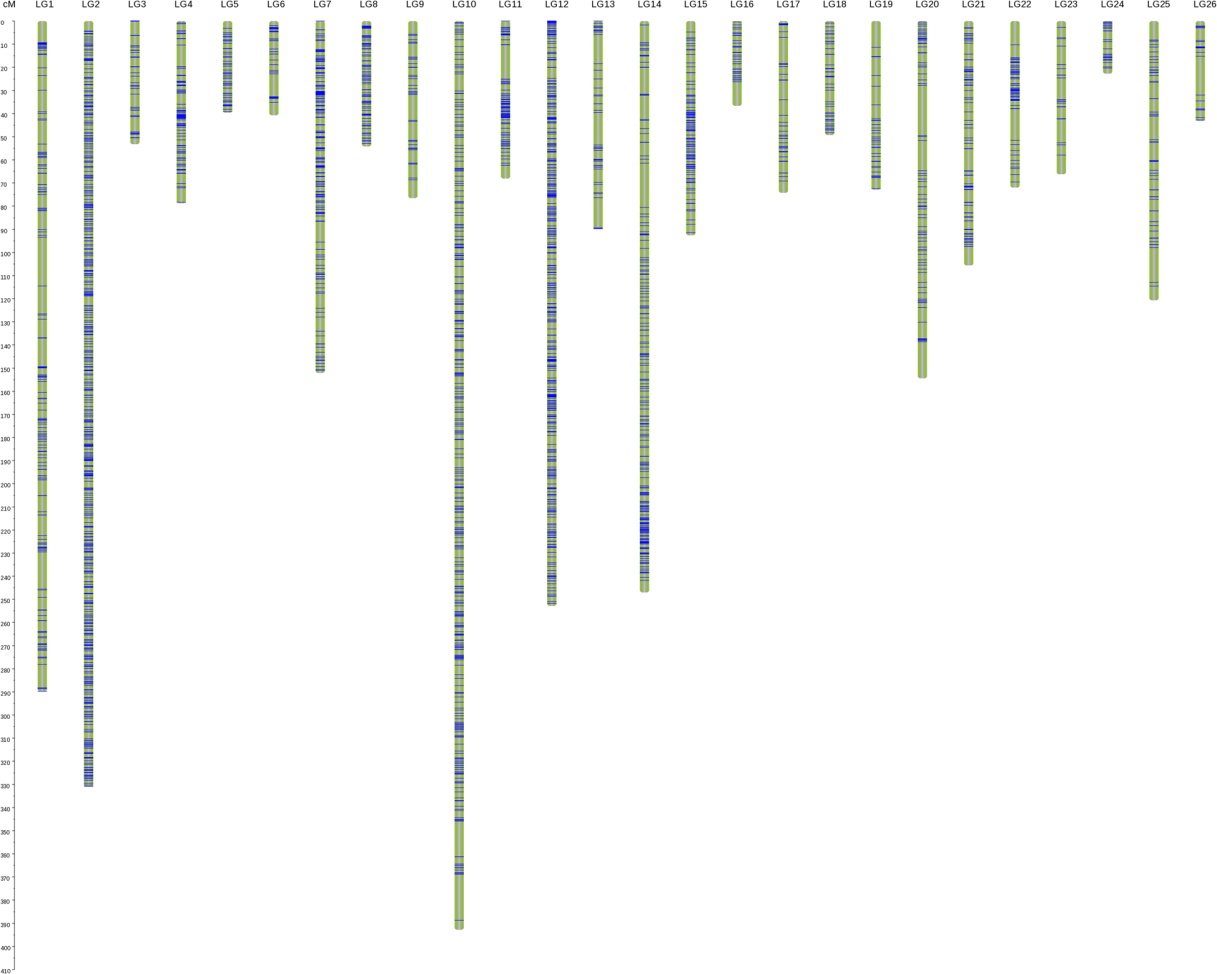
The genetic map of *G.barbadense*

The genetic map of this population was constructed using the Sea Island cotton reference genome [35]. The genetic map contained 3557 SNPs, with a coverage of 3076.23 cM and an average distance between adjacent markers of 1.05 cM (Fig. 1). The A subgenome was 1919.03 cM in length and contained 2500 markers; and the D subgenome was 1157.2 cM in length and contained 1057 markers. Thus, the A subgenome exceeded the D subgenome in map length and number of markers. The largest linkage group was LG10, and it contained 372 SNPs and spanned 392.76 cM. The shortest linkage group was LG24, and it contained 42 SNPs and spanned 22.66 cM. The average gap distance between linkage groups was 1.09 cM. The mean distance was 1–2.5 cM in 10 of 26 linkage groups and < 1 cM in the other 16 (Table 5).

**Table 5:** The SNP distribution of A and D subgroups

	Number	Length(cM)
A subgenome	2500	1919.03
D subgenome	169	1133.53
The length of the map	3569	3052.56
The average between adjacent markers		1.05
The largest LG (LG10)	372	392.76
The shortest LG (LG24)	42	22.66
The most marker(LG2)	550	330.91
The smallest (LG23)	29	66.22

### Comparisons of linkage groups with the *G. hirsutum* reference genome

The 3557 SNP markers for the linkage group were analyzed by comparing the polymorphic markers using Scaffold version, with the polymorphic marker progeny genotype of *G. hirsutum* (Table 6) using a similarity > 85% as a standard.

**Table 6:** The results of comparision between linkage groups and reference genome of *G.hirstumn* TM-1

#LG	Snp_num	Blast_Snp_num	Chr	Blast_chr_num	Blast_chr_ratio
LG2	550	408	A1	408	100.00
LG3	56	44	D05	44	100.00
LG4	126	105	D08	105	100.00
LG5	58	49	A02	49	100.00
LG8	119	19	A08	19	100.00
LG9	42	27	A6	27	100.00
LG11	117	97	A13	97	100.00
LG12	479	458	A1	458	100.00
LG13	51	38	A1	38	100.00
LG14	279	240	A10	240	100.00
LG15	129	120	A6	120	100.00
LG16	39	31	D11	31	100.00
LG17	45	34	A02	34	100.00
LG18	50	28	D13	28	100.00
LG19	38	30	D6	30	100.00
LG21	131	65	D03	65	100.00
LG23	29	13	D08	13	100.00
LG24	42	29	A07	29	100.00
LG25	68	43	A10	43	100.00
LG26	34	22	D02	22	100.00
LG10	372	327	A03	326	99.69
LG7	287	269	A08	268	99.63
LG20	88	70	A12	69	98.57
LG22	85	75	D11	67	89.33
LG6	50	38	D08	25	65.79
LG1	193	142	D07	85	59.86

The results showed that 81.8% of the SNP markers in the linkage group can be assigned to chromosomes. There were 20 linkage groups that could each be found only in one corresponding chromosome. The linkage groups LG2, LG12 and LG13 were assigned to A01, and LG5 and LG17, LG9 and LG15, LG14 and LG25, and LG4 and LG23 were assigned to A02, A06, A10 and D08, respectively. Twelve linkage groups were assigned to the A subgroup, and 8 were assigned to the D subgroup. Five linkage groups (LG6, 7, 10, 20 and 22) were assigned to two chromosomes. LG7 and LG10 were assigned to the A subgroup, whereas one part of LG6, LG20 and LG22 was assigned to the A subgroup and the other was assigned to the D subgroup, with the A subgroup presenting more markers than the D subgroup. LG1 was assigned to six chromosomes: A05, A09, A13, D05, D07 and D11.

Nineteen chromosomes contained linkage groups, of which 11 and 8 were in subgroup A and D, respectively. Chromosomes A04 and 11 and D1, 4, 9, 10 and 12 were not assigned any linkage groups. The map constructed in this study covers 73.1% of 26 chromosomes, indicating that the quality of the constructed map was good and the subsequent traits were reliable.

### QTL identification of fiber quality and lint yield traits

**Fig. 2:**
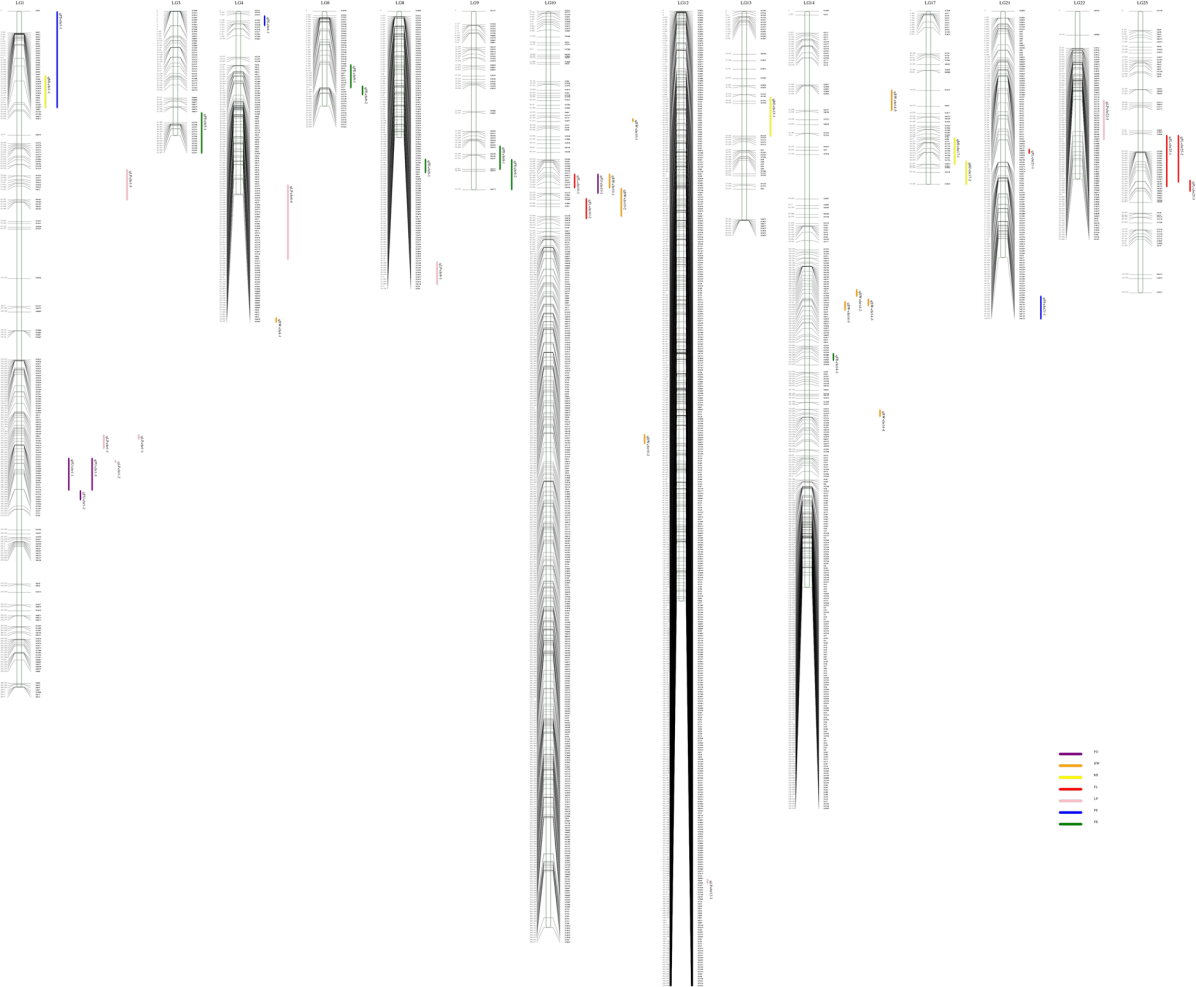
QTL associated with fiber quality and lint yield traits. Footnote: Violet: FU, Brown: BW, Yellow: MIC, Red: FL, Pink: LP, Blue: FS, Green: FE

A total of 42 QTLs associated with five fiber quality traits and two lint yield traits were identified (Table 7, Fig. 2). Twenty-four QTLs associated with five fiber quality traits (FL, FE, MIC, FS and FU) were detected. The phenotypic variation explained (PVE) was 7.2–20.7%, and the confidence interval was 3.0–20.3 cM. The highest PVE was 20.7%, and it was attributed to *qFL-LG10–1*_*C*_, which was located within a range of 8.7 cM. The smallest PVE was 7.2%, and it was attributed to *qMIC-LG1–1*_*C*_, which was located within a range of 10.2 cM. *qFE-LG14–1*_*B*_ was located in a minimum confidence interval of 3.0 cM. The FL and FU were located at LG25 and LG1, respectively, and they were detected in both environments. Eighteen QTLs were associated with two lint yield traits: BW and LP. The PVE was 0.97–19.77% and the confidence interval was 1.1–11 cM. The highest PVE associated with BW was 19.77%, and it was attributed to *qBW-LG10–2*_*A*_, which was located within a range of 9.2 cM. The smallest PVE associated with BW was 0.97%, and it was attributed to *qBW-LG14–1*_*A*_, which was located within a range of 3.1 cM.

**Table 7:** QTLs for fiber quality in the Island cotton RIL population according to *G. barbadense* scaffold

Trait	QTL	Env	LOD_Peak	LOD_Peak_Pos(cM)	Left_Marker	Right_Makrer	99%CI(cM)	Additive_effect	R2(%)
FL	*qFL-LG25–1*_*A*_	2013	3.91	60.71	LG25-M366	LG25-M4370	56.9–65.8	0.86	10.96
	*qFL-LG25–2*_*B*_	2014	3.42	60.71	LG25-M366	LG25-M4367	57–64.5	0.84	9.76
	*qFL-LG25–3*_*B*_	2014	3.45	67.81	LG25-M2503	LG25-M4860	64.5–70.4	0.97	10.95
	*qFL-LG10–1*_*C*_	2015	8.67	71.11	LG10-M5173	LG10-M4596	66.8–75.5	−0.7	20.70
	*qFL-LG10–2*_*C*_	2015	4.57	82.1	LG10-M1179	LG10-M4634	81–87.9	−0.54	12.35
	*qFL-LG21–1*_*C*_	2015	5.48	39.21	LG21-M4757	LG21-M4005	38.6–41.7	−1.04	13.16
FE	*qFE-LG8-1*_*A*_	2013	3.66	22.81	LG08-M3281	LG08-M899	20.3–24.1	0.55	9.50
	*qFE-LG9–1*_*B*_	2014	3.92	55.71	LG9-M4196	LG9-M4193	55.2–61.8	0.24	10.11
	*qFE-LG9–2*_*B*_	2014	4.73	69.61	LG9-M4327	LG9-M4273	61.8–74.6	0.28	13.05
	*qFE-LG14–1*_*B*_	2014	3.5	146.21	LG14-M2518	LG14-M2842	144.8–147.8	0.49	9.05
	*qFE-LG3-1*_*C*_	2015	3.72	47.21	LG03-M4475	LG03-M2107	41.2–50.4	−0.22	9.58
	*qFE-LG6–1*_*C*_	2015	4.78	12.11	LG6-M1946	LG6-M277	5.3–16.9	0.25	10.74
	*qFE-LG6–2*_*C*_	2015	3.83	19.91	LG6-M278	LG6-M3770	16.9–22.3	0.23	9.10
MIC	*qMIC-LG13-1*_*B*_	2014	3.47	37.71	LG13-M3742	LG13-M1129	36.7–41.4	0.13	10.11
	*qMIC-LG1–1*_*C*_	2015	3.25	14.21	LG1-M600	LG1-M1483	10.1–20.3	0.12	7.20
	*qMIC-LG17–1*_*C*_	2015	3.32	54.91	LG17-M281	LG17-M592	54.5–60.6	−0.12	8.04
	*qMIC-LG17–2*_*C*_	2015	6.29	65.71	LG17-M1705	LG17-M1602	62.3–73.2	−0.17	14.74
FS	*qFS-LG21–1*_*A*_	2013	3.38	96.51	LG21-M2558	LG21-M4135	93.1–103.5	1.36	8.86
	*qFS-LG1–1*_*B*_	2014	3.92	12.71	LG1-M587	LG1-M1483	2–22.3	1.6	10.42
	*qFS-LG4-1*_*C*_	2015	4.84	4.21	LG04-M1919	LG04-M3228	1.2–4.7	−1.02	10.11
FU	*qFU-LG1–1*_*A*_	2013	3.68	187.1	LG1-M110	LG1-M3714	184.2–191.8	−0.64	11.53
	*qFU-LG1–2*_*A*_	2013	3.34	195.81	LG1-M3714	LG1-M562	193.6–196.7	−1.15	13.44
	*qFU-LG1–3*_*B*_	2014	3.83	187.61	LG1-M110	LG1-M3714	184.2–191.8	−0.64	11.82
	*qFU-LG10–1*_*C*_	2015	4.94	71.11	LG10-M5173	LG10-M1175	66.3–75.8	−0.44	12.67
BW	*qBW-LG10–1*_*A*_	2013	4.71	69.31	LG10-M5173	LG10-M4596	67–73.4	−0.18	12.5
	*qBW-LG10–2*_*A*_	2013	7.65	78.81	LG10-M4596	LG10-M3158	76.3–85.5	−0.24	19.77
	*qBW-LG14–1*_*A*_	2013	0.44	117.91	LG14-M4633	LG14-M214	117.8–121.3	−0.13	0.97
	*qBW-LG10-3*_*B*_	2014	3.27	46.51	LG10-M976	LG10-M5057	45.5–46.7	−0.17	8.92
	*qBW-LG14–2*_*B*_	2014	4.59	112.61	LG14-M4361	LG14-M2564	110.1–113.2	−0.37	13.55
	*qBW-LG14–3*_*B*_	2014	6.1	117.81	LG14-M3255	LG14-M516	116.8–118.9	−0.41	15.58
	*qBW-LG14-4*_*B*_	2014	3.17	171.81	LG14-M1031	LG14-M978	170.8–172.7	0.29	9.76
	*qBW-LG14-5*_*B*_	2014	4.71	182.31	LG14-M2899	LG14-M4767	33–37.6	0.45	17.65
	*qBW-LG4-1*_*C*_	2015	4.00	35.91	LG04-M821	LG04-M190	77.3–81.6	0.24	10.48
	*qBW-LG10-4*_*C*_	2015	3.88	78.81	LG10-M4126	LG10-M1780	164.3–168.1	−0.52	11.26
LP	*qLP-LG1–1*_*A*_	2013	2.63	165.11	LG1-M416	LG1-M1476	174.5–179	0.1	6.95
	*qLP-LG1–2*_*A*_	2013	4.1	175.71	LG1-M111	LG1-M5042	184.5–185.6	0.1	10.39
	*qLP-LG1–3*_*A*_	2013	3.53	185.41	LG1-M1139	LG1-M4143	68.4–78.1	0.02	9.28
	*qLP-LG4-1*_*A*_	2013	3.24	74.11	LG04-M622	LG04-M620	43.9–54.9	−0.01	9.8
	*qLP-LG1-4*_*B*_	2014	5.88	49.81	LG1-M416	LG1-M112	175.2–176.9	0.01	16.15
	*qLP-LG12-1*_*B*_	2014	3.80	176.1	LG12-M4643	LG12-M1668	175.2–176.9	−0.03	10.67
	*qLP-LG22-1*_*B*_	2014	4.71	23.91	LG22-M1721	LG22-M1883	22.2–26.9	−0.1	11.25
	*qLP-LG8-1*_*C*_	2015	3.24	48.51	LG08-M2323	LG08-M4734	46.8–51.5	−0.1	8.11

Twenty-four QTLs associated with fiber quality were distributed among the 12 linkage groups: LG1 (5 QTLs), LG3 (1 QTL), LG4 (1 QTL), LG6 (2 QTLs), LG8 (1 QTL), LG9 (2 QTLs), LG10 (3 QTLs), LG13 (1 QTL), LG14 (1 QTL), LG17 (2 QTLs), LG21 (2 QTLs) and LG25 (3 QTLs). Among them, LG1 had the largest number of QTLs, followed by LG10 and LG25.

LG1 contained three traits, MIC, FS and FU. MIC was located in LG1 at M600-M1483, FS was located in LG1 at M587–M1483, and FU was located in both environments. These environments were both located in the same range in LG1 at M1010-M3714, with a confidence interval of 7.60 cM and PVE > 10%. This finding indicated that the QTL is stable.

LG10 contained two traits, FL and FU. The QTLs associated with FL were located in LG10 at M5173-M4596 and LG10 at M1179-M4634. A QTL associated with FU was located in LG10 at M5173–M1175. QTLs associated with both traits were located in LG10 at M5173-M4596, with a confidence interval of 8.7 cM.

LG25 contained QTLs for FL under two environments. Three QTLs were located in LG25 at M3606-M4370, M3606-M4367 and M2503-M4860. Of these, M2503-M4367, the common region among the three locations, was 3.84 cM, indicating that FL genes had higher relevance in this region.

Six QTLs associated with FL were detected in three environments. One QTL with a PVE of 10.96% was detected in 2013, and three QTLs were detected in 2015. The maximum PVE detected in the three environments was 20.70% as detected in 2015. Seven QTLs associated with FE were detected in the three environments. The PVE range was 9.05–13.05, indicating that the traits were quantitative and controlled by multiple genes of minor effect. Only one QTL was detected in 2013, and the rest were detected evenly between the other two environments. Four QTLs were associated with MIC, none of which were detected in 2013. One QTL was detected in 2014, and three were detected in 2015. The PVE range was 7.20–14.74% in 2015. Three QTLs associated with FS were detected in three environments, with a PVE of ~ 10%.

Eighteen QTLs were associated with lint yield, and they were distributed among seven linkage groups: LG1 (4 QTLs), LG4 (2 QTLs), LG8 (1 QTL), LG10 (4 QTLs), LG12 (1 QTL), LG14 (5 QTLs) and LG22 (1 QTL). LG14 had the largest number of QTLs, followed by LG1 and LG10.

Two linkage groups, LG10 and LG14, were associated with BW. LG10 contained four QTLs associated with BW, and they were distributed among the three environments. However, these four QTLs did not have a common confidence interval. Five QTLs were detected in LG14, and they were also distributed among the three environments. A confidence interval of 117.8–118.9 cM was detected in two environments. LG1 had four QTLs associated with LP, and they were detected in two environments. A confidence interval of 175.2–176.9 cM was detected in the two environments. The two lint yield traits were not detected in the same linkage group.

The additive effects of the 42 QTLs associated with fiber quality and lint yield traits were analyzed. The results showed that an additive effect of 22 QTLs was contributed by the paternal parent 5917, and an additive effect of 20 QTLs was contributed by the maternal parent PimaS-7.

For fiber quality traits, 24 QTLs associated with FL, FE, FS, FU and MIC indicated that an additive effect of 11 QTLs was contributed by the paternal parent 5917, and an additive effect of 13 QTLs was contributed by the maternal parent PimaS-7. Furthermore, an additive effect of QTLs associated with FU was derived from the paternal parent 5917. The only additive effect from one of seven QTLs associated with FE was contributed by the paternal parent. For FS, only one of three QTLs derived from cultivar 5917 differed significantly between the parents.

For lint yield traits, 18 QTLs were associated with BW and LP, with 11 from the paternal parent 5917 and 7 from the maternal PimaS-7. Seven of the 10 QTLs associated with BW were from the paternal 5917, and the other three were from the maternal PimaS-7. Half of the QTLs associated with LP came from each parent.

### QTL hotspots and clusters

The QTLs were not randomly distributed across chromosomes and chromosomal regions. Certain QTLs were identified in “clusters” and “hotspots,” which were defined by the presence of multiple QTLs for different or similar traits, respectively, within a 20 cM region [36–39].

In this study, one cluster and two hotspots were detected in LG1 (Table 8). The cluster possessed two QTLs pertaining to MIC and FS, which were located at 10–20 cM. One hotspot contained three QTLs related to FU, which were located at 184–196 cM. The confidence intervals of two of the three QTLs overlapped. The other hotspot contained three QTLs associated with LP, which were located at 174.5–185.6 cM.

**Table 8:** Quantitative trait locus Cluster/Hotspot for fiber quality in the Sea Island cotton

cluster/hotspot	location	QTL
LG1-cluster-1	10–20 cM	*qMIC-LG1–1, qFS-LG1–1*
LG1-hotspot-1	184-196 cM	*qFU-LG1–1, qFU-LG1–2, qFU-LG1–3*
LG1-hotspot-2	174.5–185.6 cM	*qLP-LG1–1, qLP-LG1–2, qLP-LG1–3*
LG6-hotspot-1	5-22 cM	*qFE-LG6–1, qFE-LG6–2*
LG9-hotspot-1	55-74 cM	*qFE-LG9–1, qFE-LG9–2*
LG10-hotspot-1	66-87 cM	*qFL-LG10–1, qFL-LG10–2*
LG10-hotspot-2	67–85.5 cM	*qBW-LG10–1, qBW-LG10–2*
LG10-cluster-1	66-87 cM	*qFL-LG10–1, qFL-LG10–2, qFU-LG10–1*
LG14-hotspot-1	110.1–121.3 cM	*qBW-LG14–1, qBW-LG14–2, qBW-LG14–3*
LG17-hotspot-1	54-73 cM	*qMIC-LG17–1, qMIC-LG17–2*
LG25-hotspot-1	56-70 cM	*qFL-LG25–1, qFL-LG25–2, qFL-LG25–3*

LG6 and LG9 harbored one hotspot that contained two QTLs related to FE, which were located at 5–22 cM and 55–74 cM. The two QTLs confidence intervals were adjacent.

One cluster and one hotspot were detected in LG10. The cluster harbored three QTLs related to FL and FU. These QTLs were located at 66–87 cM, and a QTL associated with the FU confidence interval was included in the FL region. One hotspot contained two QTLs pertaining to FU. We can use the overlapping region to further analyze the gene annotation to obtain useful information. One hotspot was detected in LG14. The hotspot contained three QTLs related to BW, which were located at 110.1–121.3 cM. LG17 contained one hotspot, LG17-hotspot-1, with two QTLs related to MIC, which were located at 54–73 cM. LG25 also harbored one hotspot, LG25-hotspot-1, with three QTLs related to FL, which were located at 56–70 cM. The three QTLs confidence intervals overlapped.

## Discussion

In general, the polymorphism ratio of intraspecific *G. barbadense* was 2.58% [18], which was much lower than the intraspecific polymorphism ratio (6.58%) [40] and interspecific polymorphism ratio of upland cotton (15.72%) [41]. This difference is related to the lower genetic diversity of species within the intraspecific population of *G. barbadense*. In this study, we used two Sea Island cotton cultivars as parents to determine five fiber quality traits and two lint yield traits. The phenotype results showed that significant differences did not occur in the traits except for FS and LP. Ning et al. [42] constructed a genetic map using the RIL population and mapped the QTLs for 14 yield and fiber quality traits. An analysis of the phenotypic data showed that significant differences did not occur in the five traits, which did not affect the mapping results. Li et al. [39] also used the RIL population to construct a genetic map and map traits, and two traits among the seven traits did not show significant differences, which did not affect the QTL mapping.

The RIL population is a permanent population with a higher recombination rate and higher resolution for mapping compared with the F_2_ population. In this study, the differences were small between parents, although the characteristics of the population exhibited a normal distribution, indicating that this population was suitable for the construction of a genetic map and for identifying the QTLs for fiber quality traits.

In previous studies, genetic maps were based mostly on low-throughput molecular markers. Reinisch et al. [1] constructed the first interspecific genetic map using restriction fragment length polymorphism (RFLP) markers. Since then, numerous genetic mapping studies have been performed. Genetic maps have been constructed for interspecific populations [13–16] and intraspecific populations of *G. hirsutum* [7–12]; however, few *G. barbadense* genetic maps are available. Abdelraheem et al. [17] constructed a genetic map using the RIL population from intraspecific Sea Island cotton (*G. barbadense* L.), and it was the first genetic map for *G. barbadense* with the RIL population. The map contained 247 polymorphic markers that spanned 685.7 cM, with 2.78 cM between adjacent markers. Wang et al. [18] constructed a genetic map for intraspecific Sea Island cotton that contained 52 linkage groups, of which 35 corresponded to 20 chromosomes. The total length of the genetic map was 2108.34 cM, and the average distance between adjacent markers was 6.26 cM. These genetic maps were lower in marker number, unsaturated and divided into many linkage groups.

In this study, we sequenced the *G. barbadense* RIL population using GBS technology, which has the potential for high-throughput analysis and lower genotyping error rates. The results presented here were greatly improved compared with that of previous works. First, this study had > 14-fold more markers than in the first RIL *G. barbadense* genetic map. Second, the first *G. barbadense* genetic map accounted for only 22% of the map presented in this study. Third, the previous maximum distance of 6.26 cM was shortened to 1.05 cM in this study.

Considerable work has been performed on fiber quality and lint yield trait locations. More than 1000 QTLs for fiber quality have been reported by many researchers, and 80% of which were obtained from interspecific populations [38]. Yang et al. [43] used the interspecific BC_1_ population to construct a genetic map and identify QTLs. A total of 44 QTLs for fiber quality were detected, with 20 located in the A subgroup and the remaining 24 located in the D subgroup. Four common QTLs were detected on chr5, chr7 and chr16, which contained two QTLs related to FL, FS, MIC and FU. Fang et al. [44] used the RIL population constructed using upland cotton, and 131 QTLs associated with ELO, MIC, SFC, STR, UHM, and UI were distributed on all 26 chromosomes. QTLs with marker loci were mapped at 5 cM intervals. Two chromosomes, chr16 and chr7, had the largest favorable and unfavorable breeding efforts for the four traits SFC, STR, UHM and UI, respectively. Zhang et al. [33] used a RIL population constructed with upland cotton to research BW and detected 146 QTLs for BW on 25 chromosomes across 11 environments (chromosome 8 was the exception). Sixteen QTLs were regarded as stable because they could be detected in at least three environments. Wang et al. [18] used an F_2_ of Sea Island cotton to research yield and fiber quality traits, and a total of 33 QTLs for 10 traits were detected in 1 year; however, none of these QTLs were observed in this study because of the different populations and marker types used in the studies.

In this study, we located fiber quality traits to specific regions. Forty-two QTLs associated with five fiber quality and lint yield traits were distributed among 14 linkage groups. Twenty-eight QTLs were located in the A subgenome, with the rest located in the D subgenome. LG1 exhibited the largest number of QTLs. The average genetic distance between QTL intervals was 7.8 cM, and it ranged from 3.0 cM to 20.3 cM. Most QTLs were detected in 2014. All traits except MIC were detected in the three environments.

## Conclusions

This study was the first to use the RIL population of *G. barbadense* L. and GBS to map and identify fiber quality traits. The genetic map length was 3076.23 cM, and it included 3557 SNP markers, with an average distance between adjacent markers of 1.09 cM. Forty-two QTLs were found to be associated with five fiber traits and two lint yield traits across 3 consecutive years.

## Methods

### Plant materials

The mapping population was an F_2:6_ RIL population of 143 lines generated from American cotton PimaS-7 crossed with Chinese 5917 as parents. Cultivar 5917 outperformed PimaS-7 in terms of both FL and FS and showed significant differences in FS; however, PimaS-7 outperformed 5917 in MIC. This population was planted in two replications from 2013 to 2015 in Aksu, Xinjiang Province. The test was conducted using a randomized group test design, and the grid was laid out (line length × line width, 3 m × 0.6 m) using standard field management. Cotton fiber quality traits were determined by the Cotton Fiber Quality Supervision, Inspection, and Testing Centre of the Ministry of Agriculture (HVI1000). Five fiber quality traits, including FL (mm), MIC, FS (CN/tex), FU (%) and FE (%), and two lint yields, including BW and LP (%), were examined.

### Phenotyping

Each sample containing 30 bolls from each strain was harvested when mature. Significant differences in fiber traits between the two parents were determined by one-way ANOVA. Descriptive analyses of fiber quality traits were performed using SPSS 21.0, and they included the mean, variance, kurtosis and skewness values. The broad-sense heritability was calculated using SPSS 21.0 statistical software and the following equation [45]:*H2B* (broad-sense heritability) = σ2G/(σ2G + /σ2G * E/ne + σ2E/nenr),where σ2G represents genotypic variance, σ2G * E represents the genotype * environmental variance, and σ2E is the variance of error.

### DNA quantification and qualification

Whole genomic DNA was extracted from fresh leaves of the parents and RIL populations using a plant genomic DNA extraction kit. DNA degradation and contamination were monitored on 1% agarose gels, and DNA purity was assessed using a NanoPhotometer® spectrophotometer (IMPLEN, German, CA, USA). The DNA concentration was measured using the Qubit® DNA Assay Kit and Qubit® 2.0 Fluorometer (Life Technologies, New York, CA, USA).

### Library preparation

A preliminary experiment was performed for the GBS predesign. The enzymes and restriction fragment sizes were evaluated using training data. Three criteria were considered: i) the number of tags must be suitable for the specific needs of the research project; ii) the enzymatic tags must be distributed evenly throughout the sequences to be examined; and iii) repeat tags must be avoided. These considerations improved the efficiency of GBS. To maintain sequence depth uniformity among different fragments, a tight length range was selected (~ 50 bp).

The GBS library was constructed using a predesigned scheme. For the RIL population, genomic DNA was incubated at 37 °C with *Mse*I (New England Biolabs [NEB]), T4 DNA ligase (NEB), ATP (NEB) and *Mse*I Y adapter N containing a barcode. Restriction-ligation reactions were heat-inactivated at 65 °C and then digested by the restriction enzymes *Ecol*I and *NIa*III at 37 °C. The restriction-ligation samples were purified using AgencourtAMPure XP (Beckman). PCR was performed using the purified samples mixed with the Phusion Master Mix (NEB) universal primer, index primer, and complete i5 and i7 sequences. The PCR products were purified using AgencourtAMPure XP, pooled, and then run on a 2% agarose gel. Fragments at 375–400 bp in size (with indices and adaptors) were isolated using a gel extraction kit (Qiagen). These fragments were then purified using AgencourtAMPure XP and then diluted for sequencing.

### Illumina sequencing

Paired-end sequencing was performed using the selected tags on an Illumina high-throughput sequencing platform, and it was followed by SNP genotyping and data analyses.

### Quality control

The sequences of each sample were sorted using barcodes. To ensure that the reads were reliable and devoid of artificial bias (low-quality paired reads resulting mainly from base-calling duplicates and adapter contamination) in the following analyses, raw data (raw reads) in fastq format were first processed using a series of quality control (QC) procedures performed using in-house C scripts. The QC procedures performed were as follows:

1. Remove reads with ≥10% unidentified nucleotides (N);
2. Remove reads with > 50% bases with a phred quality < 5;
3. Remove reads with > 10 nucleotides that aligned to the adapter, allowing ≤10% mismatches;
4. Remove reads containing the *Mse*I enzyme sequence.

### Mapping to the reference genome

The Burrows-Wheeler Aligner [46] was used to align the clean reads of each sample to the reference genome (settings: mem -t 4 -k 32 –M -R). Alignment files were converted to BAM files using SAM tools [47] (settings: –bS –t). If multiple read pairs had identical external coordinates, only the pair with the highest mapping quality was retained.

### Genotyping

Parent polymorphic markers were classified into eight segregation patterns (ab×cd, ef × eg, hk × hk, lm × ll, nn × np, aa×bb, ab×cc and cc × ab). For the RIL population, the segregation pattern aa×bb was chosen for genetic mapping. Prior to map construction, markers with abnormal bases or integrity < 65% or significant segregation distortion at *P* < 0.001 [33] were filtered out, missing genotype imputation was conducted based on the K-nearest neighbor algorithm [48].

### Genetic map construction

Markers were divided into bin markers using perlscript. Based on the physical position, the linkage group was divided according to the independent LOD at a value of 9–18 using the default parameters. Joinmap 4.0 software was used to construct the genetic map based on the ML method using the default parameters. Finally, we used the Kosambi function to correct genetic distance using the default parameters.

### Segregation distortion analysis

Markers with a significance of 0.001–0.05 were used to construct the high-density genetic map [33]. An SDR [34] was defined as more than three contiguous significant markers (*P* = 0.001–0.05). The SDRs distributed on the genetic map were then analyzed.

### QTL analysis

The LOD thresholds of each phenotype were determined by a permutation test using MapQTL6, with a permutation test number of 1000, a mapping step of 1.0 cM, and a significance level of *P* < 0.05. QTLs were mapped using the CIM algorithm in WinQTL Cart 2.5. The QTLs corresponding to each phenotype was determined based on the thresholds obtained from the previous permutation test.

## Abbreviations

BW: Boll weight
FE: Fiber elongation
FL: Fiber length
FS: Fiber strength
FU: Fiber uniformity
GBS: Genotyping-by-sequencing
LP: Lint percentage
MIC: Micronaire
PVE: Phenotypic variation explained
QTL: Quantitative trait locus
RIL: Recombination inbred line
SNPs: Single-nucleotide polymorphisms
